# (*E*)-3-Allyl­sulfanyl-*N*-(4-methoxy­benzyl­idene)-5-(3,4,5-trimethoxy­phen­yl)-4*H*-1,2,4-triazol-4-amine

**DOI:** 10.1107/S1600536809002645

**Published:** 2009-02-06

**Authors:** Qian-Zhu Li, Bao-An Song, Song Yang, Yu-Guo Zheng, Qing-Qing Guo

**Affiliations:** a Center for Research and Development of Fine Chemicals, Guizhou University, Key Laboratory of Green Pesticides and Agricultural Bioengineering, Ministry of Education, Guiyang 550025, People’s Republic of China; b Department of Chemistry, Bijie University, Bijie 551700, People’s Republic of China

## Abstract

The title compound, C_22_H_24_N_4_O_4_S, adopts a *trans* configuration with respect to the C=N double bond. A weak intra­molecular C—H⋯N hydrogen bond is observed between the N atom of the C=N double bond and its neighboring phenyl H atom. The crystal structure is stabilized by inter­molecular C—H⋯N hydrogen bonds and C—H⋯π inter­actions.

## Related literature

For background on the biological activity of triazole compounds, see: Bekircan & Gumrukcuoglu (2005 ▶); Ewiss *et al.* (1986 ▶); Ikizler *et al.* (1998 ▶). For hydrogen-bond motifs, see: Bernstein *et al.* (1995 ▶).
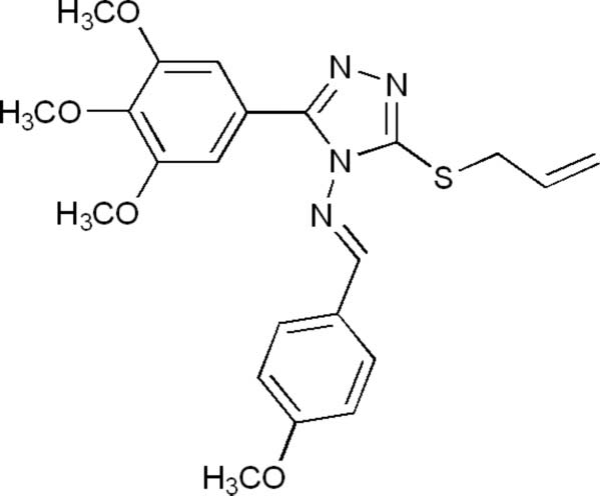


## Experimental

### 

#### Crystal data

C_22_H_24_N_4_O_4_S
*M* *_r_* = 440.51
Monoclinic, 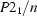

*a* = 7.9414 (12) Å
*b* = 15.043 (2) Å
*c* = 19.047 (3) Å
β = 100.385 (6)°
*V* = 2238.1 (6) Å^3^
*Z* = 4
Mo *K*α radiation
μ = 0.18 mm^−1^
*T* = 293 (2) K
0.36 × 0.30 × 0.26 mm

#### Data collection

Bruker SMART CCD area-detector diffractometer
Absorption correction: multi-scan (*SADABS*; Sheldrick, 1996 ▶) *T*_min_ = 0.936, *T*_max_ = 0.956
23323 measured reflections
3929 independent reflections
3354 reflections with *I* > 2σ(*I*)
*R*_int_ = 0.028

#### Refinement

*R*[*F* ^2^ > 2σ(*F* ^2^)] = 0.035
*wR*(*F* ^2^) = 0.103
*S* = 1.07
3929 reflections
281 parameters
H-atom parameters constrained
Δρ_max_ = 0.35 e Å^−3^
Δρ_min_ = −0.20 e Å^−3^

### 

Data collection: *SMART* (Bruker, 1997 ▶); cell refinement: *SAINT* (Bruker, 1997 ▶); data reduction: *SAINT*; program(s) used to solve structure: *SHELXS97* (Sheldrick, 2008 ▶); program(s) used to refine structure: *SHELXL97* (Sheldrick, 2008 ▶); molecular graphics: *SHELXTL* (Sheldrick, 2008 ▶); software used to prepare material for publication: *SHELXTL*.

## Supplementary Material

Crystal structure: contains datablocks I, global. DOI: 10.1107/S1600536809002645/zl2154sup1.cif

Structure factors: contains datablocks I. DOI: 10.1107/S1600536809002645/zl2154Isup2.hkl

Additional supplementary materials:  crystallographic information; 3D view; checkCIF report

## Figures and Tables

**Table 1:** Hydrogen-bond geometry (Å, °) *Cg*1 and *Cg*2 are the centroids of the C1–C6 and C13–C18 rings, respectively.

*D*—H⋯*A*	*D*—H	H⋯*A*	*D*⋯*A*	*D*—H⋯*A*
C1—H1⋯N4	0.93	2.38	2.960 (2)	120
C12—H12⋯N2^i^	0.93	2.59	3.359 (2)	141
C19—H19*A*⋯N1^ii^	0.96	2.60	3.477 (3)	152
C9—H9*A*⋯*Cg*1^iii^	0.97	2.79	3.616 (2)	143
C11—H11*A*⋯*Cg*2^iv^	0.93	2.83	3.703 (2)	158
C15—H15⋯*Cg*1^v^	0.93	2.70	3.514 (2)	147
C22—H22*C*⋯*Cg*2^vi^	0.96	2.94	3.747 (2)	143

Symmetry codes: (i) 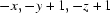; (ii) 
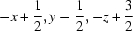
; (iii) 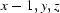; (iv) 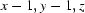; (v) 
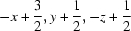
; (vi) 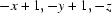.

**Fig. 1.**
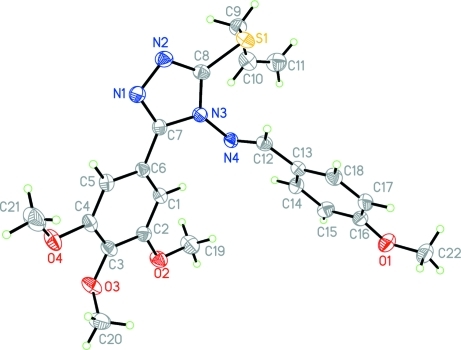
The structure of the title compound showing displacement ellipsoids drawn at the 30% probability level.

**Fig. 2.**
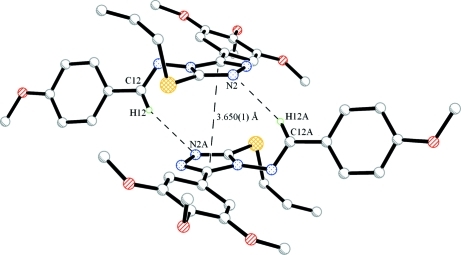
A perspective view of the R22(12) ring motif formed through the intermolecular C12—H12···N2 hydrogen bond. Dashed lines indicate C-H···N hydrogen bonds and π-π stacking interactions.

**Fig. 3.**
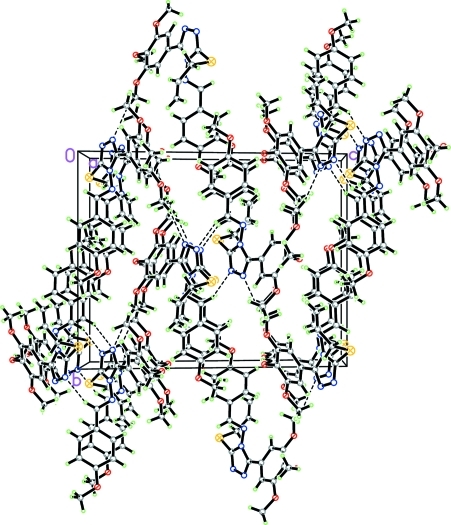
Crystal structure of the title compound viewed along the a-axis. Hydrogen bonds are shown as dashed lines.

## supplementary crystallographic information

### Comment

Triazole derivatives are of great interest in medicinal chemistry in relation to antibacterial bioactivities (Bekircan & Gumrukcuoglu, 2005; Ewiss *et al.*, 1986; Ikizler *et al.*, 1998). However, to date, only a few reports have been dedicated to the synthesis and antimicrobial activity evaluation of triazole derivatives with a 3,4,5-trimethoxyphenyl substituent. Herein, we want to report on the synthesis and structure such a compound, (*E*)-4-(4-methoxybenzylideneamino)-5-(3,4,5-trimethoxyphenyl)-4*H*-1,2,4-triazole-3-thiol.

The molecule of the title compound (Fig. 1), exists in an *E *configuration with respect to the C12=N4 double bond [1.278 (2) Å] with a N3–N4–C12–C13 torsion angle of 179.08 (13)°. The whole molecule is not planar as the dihedral angles between the triazole ring and the two phenyl rings are 25.3 (2)° and 113.8 (2)°, respectively. There is one weak intramolecular C–H···N hydrogen bond between C1 and N4 (Table 1).

In the crystal structure (Fig. 3), two neighboring molecules are linked by weak C12—H12···N2 intermolecular interactions into a centrosymmetric *R*_2_^2^(12) ring motif (Bernstein *et al.*, 1995) with two parallel trizole rings with a centroid-centroid separation of 3.650 (1) Å between them (Fig. 2). Moreover, an intermolecular C-H···N hydrogen bond (C19—H19A···N1) is also observed. The molecular packing is further stabilized by C—H···π interactions (Table 1, *Cg*1 and *Cg*2 are the centroids of the C1–C6 and C13–C18 rings, respectively).

### Experimental

A mixture of 3-bromoprop-1-ene (5 mmol) and methanol (3 mL) was added dropwise to a stirred solution of (*E*)-4-(4-methoxybenzylideneamino)-5-(3,4,5-trimethoxyphenyl)-4H-1,2,4-triazole-3-thiol (5 mmol) and sodium hydroxide (5 mmol) in water (15 mL). The resulting mixture was stirred at room temperature for 4 hours. After allowing the resulting solution to stand in air at room temperature for 2 days, colorless block-shaped crystals were formed at the bottom of the vessel on slow evaporation of the solvent. The crystals were isolated, washed with ethanol and dried.

### Refinement

H atoms were placed in calculated positions and were treated as riding on the parent C atoms with C—H = 0.93 - 0.97 Å, and with *U*_iso_(H) = 1.5 *U*_eq_(C) for methyl C atoms or *U*_iso_(H) = 1.2 *U*_eq_(C) for the other C atoms.

### Crystal data

**Table:**

C_22_H_24_N_4_O_4_S	*F*(000) = 928
*M**_r_* = 440.51	*D*_x_ = 1.307 Mg m^−^^3^
Monoclinic, *P*2_1_/*n*	Mo *K*α radiation, λ = 0.71073 Å
Hall symbol: -P 2yn	Cell parameters from 2895 reflections
*a* = 7.9414 (12) Å	θ = 2.4–27.9°
*b* = 15.043 (2) Å	µ = 0.18 mm^−^^1^
*c* = 19.047 (3) Å	*T* = 293 K
β = 100.385 (6)°	Block, colorless
*V* = 2238.1 (6) Å^3^	0.36 × 0.30 × 0.26 mm
*Z* = 4	

### Data collection

**Table:**

Bruker SMART CCD area-detector diffractometer	3929 independent reflections
Radiation source: fine-focus sealed tube	3354 reflections with *I* > 2σ(*I*)
graphite	*R*_int_ = 0.028
φ and ω scans	θ_max_ = 25.0°, θ_min_ = 1.7°
Absorption correction: multi-scan (*SADABS*; Sheldrick, 1996)	*h* = −8→9
*T*_min_ = 0.936, *T*_max_ = 0.956	*k* = −17→17
23323 measured reflections	*l* = −22→22

### Refinement

**Table:**

Refinement on *F*^2^	Secondary atom site location: difference Fourier map
Least-squares matrix: full	Hydrogen site location: inferred from neighbouring sites
*R*[*F*^2^ > 2σ(*F*^2^)] = 0.035	H-atom parameters constrained
*wR*(*F*^2^) = 0.103	*w* = 1/[σ^2^(*F*_o_^2^) + (0.0532*P*)^2^ + 0.4889*P*] where *P* = (*F*_o_^2^ + 2*F*_c_^2^)/3
*S* = 1.07	(Δ/σ)_max_ = 0.001
3929 reflections	Δρ_max_ = 0.35 e Å^−^^3^
281 parameters	Δρ_min_ = −0.20 e Å^−^^3^
0 restraints	Extinction correction: *SHELXL97* (Sheldrick, 2008), Fc^*^=kFc[1+0.001xFc^2^λ^3^/sin(2θ)]^-1/4^
Primary atom site location: structure-invariant direct methods	Extinction coefficient: 0.0132 (12)

### Special details

**Table:**

Geometry. All e.s.d.'s (except the e.s.d. in the dihedral angle between two l.s. planes) are estimated using the full covariance matrix. The cell e.s.d.'s are taken into account individually in the estimation of e.s.d.'s in distances, angles and torsion angles; correlations between e.s.d.'s in cell parameters are only used when they are defined by crystal symmetry. An approximate (isotropic) treatment of cell e.s.d.'s is used for estimating e.s.d.'s involving l.s. planes.
Refinement. Refinement of *F*^2^ against ALL reflections. The weighted *R*-factor *wR* and goodness of fit *S* are based on *F*^2^, conventional *R*-factors *R* are based on *F*, with *F* set to zero for negative *F*^2^. The threshold expression of *F*^2^ > σ(*F*^2^) is used only for calculating *R*-factors(gt) *etc*. and is not relevant to the choice of reflections for refinement. *R*-factors based on *F*^2^ are statistically about twice as large as those based on *F*, and *R*- factors based on ALL data will be even larger.

### Fractional atomic coordinates and isotropic or equivalent isotropic displacement parameters (Å^2^)

**Table:**

	*x*	*y*	*z*	*U*_iso_*/*U*_eq_	
S1	−0.32086 (5)	0.40159 (3)	0.50696 (2)	0.05036 (16)	
O2	0.53116 (15)	0.34899 (8)	0.80320 (6)	0.0566 (3)	
O4	0.63039 (17)	0.63866 (9)	0.73084 (7)	0.0647 (4)	
O1	0.27845 (18)	−0.05796 (9)	0.56221 (8)	0.0737 (4)	
N4	0.04176 (16)	0.33704 (8)	0.60779 (7)	0.0419 (3)	
N3	−0.00642 (16)	0.42733 (8)	0.59683 (7)	0.0392 (3)	
O3	0.72831 (15)	0.49408 (9)	0.81168 (6)	0.0614 (4)	
N2	−0.15075 (18)	0.54951 (9)	0.56327 (8)	0.0505 (4)	
N1	0.00133 (18)	0.57222 (9)	0.60765 (8)	0.0493 (3)	
C13	0.10497 (19)	0.20262 (10)	0.55230 (8)	0.0412 (4)	
C3	0.5743 (2)	0.49373 (11)	0.76468 (8)	0.0461 (4)	
C6	0.25403 (19)	0.49468 (10)	0.67595 (8)	0.0394 (3)	
C12	0.05405 (19)	0.29577 (10)	0.55028 (8)	0.0426 (4)	
H12	0.0303	0.3254	0.5068	0.051*	
C14	0.1270 (2)	0.15253 (11)	0.61543 (9)	0.0464 (4)	
H14	0.1020	0.1778	0.6569	0.056*	
C8	−0.1542 (2)	0.46257 (10)	0.55731 (8)	0.0431 (4)	
C7	0.0855 (2)	0.49833 (10)	0.62810 (8)	0.0398 (3)	
C1	0.3077 (2)	0.41986 (10)	0.71705 (8)	0.0415 (4)	
H1	0.2368	0.3704	0.7150	0.050*	
C2	0.4679 (2)	0.41929 (11)	0.76131 (8)	0.0435 (4)	
C5	0.3589 (2)	0.56949 (10)	0.67940 (8)	0.0443 (4)	
H5	0.3221	0.6196	0.6524	0.053*	
C4	0.5188 (2)	0.56862 (11)	0.72351 (9)	0.0463 (4)	
C15	0.1852 (2)	0.06656 (11)	0.61640 (10)	0.0517 (4)	
H15	0.2013	0.0342	0.6587	0.062*	
C16	0.2205 (2)	0.02742 (11)	0.55411 (10)	0.0518 (4)	
C18	0.1392 (2)	0.16211 (12)	0.49085 (9)	0.0517 (4)	
H18	0.1234	0.1943	0.4485	0.062*	
C10	−0.3730 (3)	0.30522 (14)	0.62529 (10)	0.0633 (5)	
H10	−0.2735	0.3199	0.6569	0.076*	
C9	−0.4510 (2)	0.37642 (12)	0.57463 (10)	0.0558 (4)	
H9A	−0.4645	0.4300	0.6014	0.067*	
H9B	−0.5639	0.3575	0.5511	0.067*	
C19	0.4213 (3)	0.27423 (13)	0.80370 (12)	0.0717 (6)	
H19A	0.4793	0.2294	0.8349	0.108*	
H19B	0.3912	0.2507	0.7562	0.108*	
H19C	0.3194	0.2922	0.8203	0.108*	
C17	0.1965 (2)	0.07488 (12)	0.49092 (10)	0.0561 (5)	
H17	0.2184	0.0488	0.4492	0.067*	
C21	0.5991 (3)	0.70808 (15)	0.68073 (15)	0.0924 (8)	
H21A	0.6853	0.7531	0.6925	0.139*	
H21B	0.4884	0.7333	0.6816	0.139*	
H21C	0.6021	0.6852	0.6339	0.139*	
C11	−0.4301 (3)	0.22612 (15)	0.62922 (12)	0.0764 (6)	
H11A	−0.5292	0.2083	0.5987	0.092*	
H11B	−0.3724	0.1865	0.6626	0.092*	
C20	0.8754 (3)	0.47977 (17)	0.78013 (13)	0.0797 (6)	
H20A	0.9762	0.4808	0.8165	0.120*	
H20B	0.8829	0.5258	0.7459	0.120*	
H20C	0.8664	0.4231	0.7566	0.120*	
C22	0.3150 (3)	−0.10321 (14)	0.50050 (15)	0.0858 (8)	
H22A	0.3545	−0.1623	0.5135	0.129*	
H22B	0.4019	−0.0714	0.4818	0.129*	
H22C	0.2129	−0.1063	0.4648	0.129*	

### Atomic displacement parameters (Å^2^)

**Table:**

	*U*^11^	*U*^22^	*U*^33^	*U*^12^	*U*^13^	*U*^23^
S1	0.0433 (3)	0.0626 (3)	0.0420 (3)	−0.00628 (19)	−0.00102 (17)	0.00451 (18)
O2	0.0536 (7)	0.0571 (7)	0.0529 (7)	−0.0027 (6)	−0.0065 (5)	0.0096 (6)
O4	0.0612 (8)	0.0609 (8)	0.0682 (8)	−0.0265 (6)	0.0013 (6)	−0.0029 (6)
O1	0.0718 (9)	0.0497 (7)	0.0989 (11)	0.0117 (6)	0.0139 (8)	−0.0119 (7)
N4	0.0437 (7)	0.0341 (7)	0.0443 (7)	−0.0016 (5)	−0.0015 (6)	0.0014 (5)
N3	0.0391 (7)	0.0357 (6)	0.0410 (7)	−0.0017 (5)	0.0026 (5)	0.0035 (5)
O3	0.0438 (7)	0.0890 (9)	0.0478 (7)	−0.0136 (6)	−0.0018 (5)	−0.0031 (6)
N2	0.0465 (8)	0.0451 (8)	0.0576 (9)	0.0013 (6)	0.0032 (6)	0.0085 (6)
N1	0.0475 (8)	0.0402 (7)	0.0582 (9)	−0.0013 (6)	0.0036 (7)	0.0031 (6)
C13	0.0378 (8)	0.0434 (8)	0.0413 (8)	−0.0037 (6)	0.0038 (6)	−0.0012 (7)
C3	0.0399 (9)	0.0610 (10)	0.0365 (8)	−0.0068 (7)	0.0046 (7)	−0.0069 (7)
C6	0.0382 (8)	0.0426 (8)	0.0378 (8)	−0.0028 (6)	0.0076 (6)	−0.0048 (6)
C12	0.0397 (8)	0.0447 (8)	0.0421 (9)	−0.0025 (7)	0.0042 (7)	0.0056 (7)
C14	0.0513 (9)	0.0446 (9)	0.0451 (9)	−0.0001 (7)	0.0137 (7)	0.0000 (7)
C8	0.0408 (9)	0.0453 (9)	0.0423 (9)	−0.0010 (7)	0.0045 (7)	0.0076 (7)
C7	0.0419 (8)	0.0371 (8)	0.0406 (8)	−0.0032 (6)	0.0083 (7)	0.0002 (6)
C1	0.0415 (8)	0.0427 (8)	0.0396 (8)	−0.0062 (6)	0.0056 (7)	−0.0025 (6)
C2	0.0440 (9)	0.0486 (9)	0.0376 (8)	0.0000 (7)	0.0063 (7)	−0.0020 (7)
C5	0.0493 (9)	0.0403 (8)	0.0434 (9)	−0.0051 (7)	0.0087 (7)	−0.0021 (7)
C4	0.0448 (9)	0.0505 (9)	0.0445 (9)	−0.0136 (7)	0.0101 (7)	−0.0086 (7)
C15	0.0523 (10)	0.0467 (9)	0.0575 (10)	0.0028 (8)	0.0141 (8)	0.0081 (8)
C16	0.0399 (9)	0.0437 (9)	0.0707 (12)	−0.0015 (7)	0.0068 (8)	−0.0087 (8)
C18	0.0538 (10)	0.0591 (10)	0.0404 (9)	−0.0002 (8)	0.0038 (7)	−0.0009 (7)
C10	0.0613 (12)	0.0763 (13)	0.0538 (11)	−0.0111 (10)	0.0142 (9)	−0.0017 (9)
C9	0.0467 (10)	0.0563 (10)	0.0668 (12)	−0.0027 (8)	0.0169 (9)	−0.0012 (9)
C19	0.0762 (14)	0.0586 (11)	0.0720 (13)	−0.0088 (10)	−0.0088 (10)	0.0201 (10)
C17	0.0496 (10)	0.0647 (11)	0.0531 (11)	0.0005 (8)	0.0068 (8)	−0.0199 (9)
C21	0.0758 (15)	0.0691 (14)	0.124 (2)	−0.0331 (12)	−0.0033 (14)	0.0223 (14)
C11	0.0755 (14)	0.0708 (14)	0.0833 (15)	0.0001 (11)	0.0154 (12)	0.0125 (11)
C20	0.0458 (11)	0.1008 (17)	0.0890 (16)	0.0021 (11)	0.0027 (11)	−0.0133 (13)
C22	0.0673 (14)	0.0626 (13)	0.128 (2)	0.0019 (10)	0.0205 (14)	−0.0384 (13)

### Geometric parameters (Å, °)

**Table:**

S1—C8	1.7477 (16)	C1—H1	0.9300
S1—C9	1.8312 (18)	C5—C4	1.391 (2)
O2—C2	1.3649 (19)	C5—H5	0.9300
O2—C19	1.424 (2)	C15—C16	1.397 (2)
O4—C4	1.3676 (19)	C15—H15	0.9300
O4—C21	1.406 (3)	C16—C17	1.383 (3)
O1—C16	1.364 (2)	C18—C17	1.389 (2)
O1—C22	1.433 (3)	C18—H18	0.9300
N4—C12	1.278 (2)	C10—C11	1.280 (3)
N4—N3	1.4164 (17)	C10—C9	1.499 (3)
N3—C7	1.3681 (19)	C10—H10	0.9300
N3—C8	1.3810 (19)	C9—H9A	0.9700
O3—C3	1.3795 (19)	C9—H9B	0.9700
O3—C20	1.423 (2)	C19—H19A	0.9600
N2—C8	1.313 (2)	C19—H19B	0.9600
N2—N1	1.3863 (19)	C19—H19C	0.9600
N1—C7	1.319 (2)	C17—H17	0.9300
C13—C18	1.389 (2)	C21—H21A	0.9600
C13—C14	1.403 (2)	C21—H21B	0.9600
C13—C12	1.457 (2)	C21—H21C	0.9600
C3—C2	1.397 (2)	C11—H11A	0.9300
C3—C4	1.398 (2)	C11—H11B	0.9300
C6—C1	1.393 (2)	C20—H20A	0.9600
C6—C5	1.394 (2)	C20—H20B	0.9600
C6—C7	1.478 (2)	C20—H20C	0.9600
C12—H12	0.9300	C22—H22A	0.9600
C14—C15	1.372 (2)	C22—H22B	0.9600
C14—H14	0.9300	C22—H22C	0.9600
C1—C2	1.394 (2)		
			
C8—S1—C9	100.95 (8)	C16—C15—H15	119.9
C2—O2—C19	117.02 (13)	O1—C16—C17	125.13 (17)
C4—O4—C21	118.08 (15)	O1—C16—C15	114.57 (17)
C16—O1—C22	117.95 (17)	C17—C16—C15	120.29 (16)
C12—N4—N3	113.56 (12)	C17—C18—C13	121.84 (16)
C7—N3—C8	105.79 (12)	C17—C18—H18	119.1
C7—N3—N4	125.15 (12)	C13—C18—H18	119.1
C8—N3—N4	128.97 (12)	C11—C10—C9	126.3 (2)
C3—O3—C20	115.13 (14)	C11—C10—H10	116.8
C8—N2—N1	107.50 (13)	C9—C10—H10	116.8
C7—N1—N2	108.17 (13)	C10—C9—S1	112.37 (13)
C18—C13—C14	118.26 (15)	C10—C9—H9A	109.1
C18—C13—C12	119.68 (14)	S1—C9—H9A	109.1
C14—C13—C12	122.03 (14)	C10—C9—H9B	109.1
O3—C3—C2	119.41 (15)	S1—C9—H9B	109.1
O3—C3—C4	120.93 (15)	H9A—C9—H9B	107.9
C2—C3—C4	119.56 (15)	O2—C19—H19A	109.5
C1—C6—C5	120.38 (15)	O2—C19—H19B	109.5
C1—C6—C7	121.86 (13)	H19A—C19—H19B	109.5
C5—C6—C7	117.75 (14)	O2—C19—H19C	109.5
N4—C12—C13	120.54 (14)	H19A—C19—H19C	109.5
N4—C12—H12	119.7	H19B—C19—H19C	109.5
C13—C12—H12	119.7	C16—C17—C18	118.84 (16)
C15—C14—C13	120.48 (15)	C16—C17—H17	120.6
C15—C14—H14	119.8	C18—C17—H17	120.6
C13—C14—H14	119.8	O4—C21—H21A	109.5
N2—C8—N3	109.43 (13)	O4—C21—H21B	109.5
N2—C8—S1	124.92 (12)	H21A—C21—H21B	109.5
N3—C8—S1	125.65 (12)	O4—C21—H21C	109.5
N1—C7—N3	109.08 (14)	H21A—C21—H21C	109.5
N1—C7—C6	124.59 (14)	H21B—C21—H21C	109.5
N3—C7—C6	126.32 (13)	C10—C11—H11A	120.0
C6—C1—C2	119.90 (14)	C10—C11—H11B	120.0
C6—C1—H1	120.1	H11A—C11—H11B	120.0
C2—C1—H1	120.1	O3—C20—H20A	109.5
O2—C2—C1	123.92 (14)	O3—C20—H20B	109.5
O2—C2—C3	115.99 (14)	H20A—C20—H20B	109.5
C1—C2—C3	120.09 (15)	O3—C20—H20C	109.5
C4—C5—C6	119.56 (15)	H20A—C20—H20C	109.5
C4—C5—H5	120.2	H20B—C20—H20C	109.5
C6—C5—H5	120.2	O1—C22—H22A	109.5
O4—C4—C5	123.97 (16)	O1—C22—H22B	109.5
O4—C4—C3	115.52 (15)	H22A—C22—H22B	109.5
C5—C4—C3	120.51 (14)	O1—C22—H22C	109.5
C14—C15—C16	120.28 (16)	H22A—C22—H22C	109.5
C14—C15—H15	119.9	H22B—C22—H22C	109.5

### Hydrogen-bond geometry (Å, °)

**Table:**

*D*—H···*A*	*D*—H	H···*A*	*D*···*A*	*D*—H···*A*
C1—H1···N4	0.93	2.38	2.960 (2)	120
C12—H12···N2^i^	0.93	2.59	3.359 (2)	141
C19—H19A···N1^ii^	0.96	2.60	3.477 (3)	152
C9—H9A···Cg1^iii^	0.97	2.79	3.616 (2)	143
C11—H11A···Cg2^iv^	0.93	2.83	3.703 (2)	158
C15—H15···Cg1^v^	0.93	2.70	3.514 (2)	147
C22—H22C···Cg2^vi^	0.96	2.94	3.747 (2)	143

Symmetry codes: (i) −*x*, −*y*+1, −*z*+1; (ii) −*x*+1/2, *y*−1/2, −*z*+3/2; (iii) *x*−1, *y*, *z*; (iv) *x*−1, *y*−1, *z*; (v) −*x*+3/2, *y*+1/2, −*z*+1/2; (vi) −*x*+1, −*y*+1, −*z*.
